# The biology of medicinal resource substitution in *Salvia*

**DOI:** 10.1186/s13020-021-00548-6

**Published:** 2021-12-23

**Authors:** Ning Cui, Tiezhu Chen, Baosheng Liao, Jiang Xu, Xiwen Li

**Affiliations:** 1 Institute of Chinese Materia Medica, China Academy of Chinese Medical Sciences, Beijing, 100700 China; 2 Central Laboratory, Shandong Academy of Chinese Medicine, Jinan, 250014 China; 3 Sichuan Provincial Key Laboratory of Quality and Innovation Research of Chinese Materia Medica, Sichuan Academy of Traditional Chinese Medicine Sciences, Chengdu, 610041 China; 4 Key Laboratory of Quality Evaluation of Chinese Medicine of the Guangdong Provincial Medical Products Administration, The Second Clinical College, Guangzhou University of Chinese Medicine, Guangzhou, China

**Keywords:** Alternative species, Ecology, Medicinal resource substitution, Phylogeny, *Salvia*

## Abstract

**Background:**

The decrease of wild reserves and the sharp increase of market demand have led to resource substitution, but it is still not clear how to discover medicinal alternative resources. Here we reveal the biology of medicinal resource substitution in the case of *Salvia*.

**Methods:**

A hypothesis was put forward that phylogeny and ecology were the main factors which determined alternative species selection. Phylogenetic analysis was performed based on chloroplast genomes. Spatial climatic pattern was assessed through three mathematical models.

**Results:**

*Salvia miltiorrhiza* and alternative species were mainly located in Clade 3 in topology, and their growth environment was clustered into an independent group 3 inferred from principal component analysis. Correlation and Maxent major climate factor analyses showed that the ecological variations within each lineage were significantly smaller than the overall divergent between any two lineages. Mantel test reconfirmed the inalienability between phylogeny and ecology (*P* = 0.002). Only the species that are genetically and ecologically related to *S. miltiorrhiza* can form a cluster with it.

**Conclusions:**

Phylogenetic relationship and geographical climate work together to determine which species has the potential to be selected as substitutes. Other medicinal plants can learn from this biology towards developing alternative resources.

**Supplementary Information:**

The online version contains supplementary material available at 10.1186/s13020-021-00548-6.

## Introduction

Traditional Chinese medicine (TCM) has now become popular worldwide due to its significant effect, cost-effectiveness and low incidence of side effects [1]. The increasing demand for TCM has led to reduction of wild resources and medicinal resource substitution [2, 3]. In general, species with large natural reserves or those that can be easily cultivated with similar efficacy are often chosen as substitutes, which occur in many TCMs. For instance, since 1993, when the State Department in China issued a notice banning the trade of rhino horn [4], it has been removed from Chinese Pharmacopoeia and replaced by water buffalo horn [5].

As an important TCM, Salviae miltiorrhizae Radix et Rhizoma (Danshen in Chinese) has been used for more than 2000 years to promote blood circulation and relieve pain. Because of its anti-thrombotic properties, Danshen is often used to treat cardiovascular disorders [6]. However, the official raw material of Danshen in China is the root and rhizome of one single species of *Salvia miltiorrhiza* Bge. [7]. There are some problems in the cultivation of *S. miltiorrhiza*, such as continuous cropping obstacle and germplasm decline after domestication [8, 9]. The resource supply of *S. miltiorrhiza* cannot fully meet people’s needs [10]. In this case, more than 20 congeneric species are used as substitutes for *S. miltiorrhiza* (named as *S. miltiorrhiza* substitutes, SMSs) by local folks [11] because of their similar pharmacological effects [12]. For instance, Nandanshen, the root of *S. bowleyana* Dunn, was used as Danshen in history in some areas of Zhejiang, Jiangxi and Anhui provinces in China [11]. Simultaneously, many other *Salvia* species exist. Their application part is the whole plant, and their application or efficacy is distinct from Danshen and thus could not be eligible substitutes (named as non-substitutes for *S. miltiorrhiza*, nSMSs). For example, *S. plebeia* principally has anti-inflammatory, antioxidative, antibacterial and antiviral activities, not including anti-thrombotic effects [13]. *S. leucantha* has antibacterial activity and is typically used as an ornamental flower [14]. Basing on the classification of Xiao et al. [12], we suggested that *Salvia* species could be divided into three groups, *S. miltiorrhiza*, *S. miltiorrhiza* substitutes (SMSs) and non-substitutes for *S. miltiorrhiza* (nSMSs; Table 1) according to whether species could be used as substitutes for *S. miltiorrhiza* or not. In this regard, we speculated about the factors that could determine which species could be used as *S. miltiorrhiza* substitutes. Thus far, no in-depth attempts have been made to study vital determinants, leading to ambiguity in the development of alternative medicinal resources.

**Table 1:** Practical applications of SMSs and nSMSs in this study

Species	Practical application	Newly collected in this study?
*S. japonica*	The whole plant was used to treat cough, sore, rheumatism, etc [15]	No
*S. officinalis*	The whole plant was used as spices or to treat indigestion [16]	No
*S. deserta*	The plant root was used as *S. miltiorrhiza* alternatives in folk and to treat cardiovascular diseases [17]	Yes
*S. hispanica*	The whole plant was used as spices, bactericide, anthelmintic or for regulating the central nervous system [18]	No
*S. leucantha*	The whole plant was used as ornamental flower or for regulating the central nervous system [19]	Yes
*S. pansamalensis*	ditto [20]	Yes
*S. plebeia*	The whole plant was used to treat fall, tracheitis, cervicitis, etc [21]	No
*S. roborowskii*	The whole plant was used to clear liver, clear eyes and relieve pains [22]	No
*S. miltiorrhiza*	The plant root was used as TCM Danshen to treat cardiovascular diseases [23]	No
*S. yunnanensis*	The plant root was used as *S. miltiorrhiza* alternatives in folk medicine to treat cardiovascular diseases [24]	No
*S. prattii*	ditto [25]	No
*S. bulleyana*	ditto [26]	No
*S. digitaloides*	ditto [27]	Yes
*S. przewalskii*	ditto [28]	No

Pharmaphylogeny, founded by Prof. Pei-gen Xiao in the 1980s, is an emerging discipline that focuses on the intrinsic correlation of molecular phylogeny, chemical constituents and therapeutic efficacy [29]. The theory highly emphasizes the importance of phylogeny in substitution, that is, related species are speculated to possess similar biological activities and clinical efficacy. However, Xiao [30] himself also thought that some plant medicines whose related species are found in China have different pharmacological effects from the target medicine; hence, there could be other factors, apart from phylogenetics, influencing whether candidate species could be used as substitutes or not.

In this study, we attempted to reveal the biology of medicinal resource substitution in *Salvia*. Basing on the formation and scientific elucidation of daodi medicinal materials [31, 32], we hypothesized that phylogenetic relationship and geographical climate are the main factors that determine which *Salvia* species has the potential to be selected as substitutes for *S. miltiorrhiza*. This study aimed to (i) reveal key influential factors in *S. miltiorrhiza* substitution; (ii) explore the joint action of phylogeny and ecology to acquire efficacy homogeneity and diversity of *Salvia* species; and (iii) provide a screening model for herbal substitution and new medicinal resource discovery. This paper is the first report to reveal the relationship between TCM and its substitution and attempt to explain the underlying causes from the perspective of phylogeny and ecology.

## Materials and methods

### Plant material and DNA extraction

*Salvia* plants were selected referring to previous studies [21, 33–36] by considering extraordinary characteristics, including genetic diversity, global distribution and medicinal significance for treating cardiovascular diseases. The sample selection in this study was primarily based on the article by Xiao et al. [12], and few other species with definite applications were also supplemented in consideration of the species distribution and systematic taxonomy of *Salvia*. Fresh leaves of four *Salvia* species (*S. deserta*, *S. leucantha*, *S. pansamalensis* and *S. digitaloides*), covering all of three global distribution centers, Central and South America, central Asia/Mediterranean and eastern Asia [37], were sampled, and their collection information was listed in Table 2. Voucher specimens were deposited in the Institute of Chinese Materia Medica, China Academy of Chinese Medical Sciences.

**Table 2:** Collection information of four *Salvia* species sequenced in this study

	Species	Collection region	Accession number
1	*S. deserta*	Fukang County, Fukang City, Xinjiang Uygur Autonomous Region, China	MT156378
2	*S. leucantha*	Guanajuato, Mexico	MT156367
3	*S. pansamalensis*	Chiapas, Mexico	MT156368
4	*S. digitaloides*	Shangri-La County, Diqing Tibetan Autonomous Prefecture, Yunnan Province, China	MT156376

Total genomic DNA was extracted from 100 mg of the silica-dried leaf by using a Dneasy Plant MiniKit (Qiagen, CA, USA) according to the manufacturer’s instructions. The quantity and quality of genomic DNA was examined using ND-2000 spectrometer (ThermoFisher Scientific, Wilmington, DE, USA) and 0.8% agarose gel electrophoresis.

### Sequencing, chloroplast genome assembly and annotation

The chloroplast (cp) genome of *S. plebeia* (NC050929) was assembled in our previous study [21]. Taking this work as a guidance, the DNA sample pre-treatment, whole genome sequencing, cp genome assembly, junction validation, and cp genome annotation were performed in turn. Four cp genomes were submitted to the NCBI database (www.ncbi.nlm.nih.gov) with GenBank accession numbers listed in Table 2. The physical maps of cp genomes were produced with Organellar Genome DRAW [38] (http://ogdraw.mpimp-golm.mpg.de/).

### Genome comparative analyses

In addition to the four newly sequenced cp genomes, the 10 following available cp genome sequences of *Salvia* were downloaded from the NCBI database: *S. japonica* (NC035233), *S. officinalis* (NC038165), *S. hispanica* (NC046838), *S. plebeia* (NC050929), *S. miltiorrhiza* (NC020431), *S. yunnanensis* (MK944405), *S. prattii* (MK944407), *S. roborowskii* (MK944406), *S. bulleyana* (NC041092) and *S. przewalskii* (NC041091). The multiple sequence alignment of the 14 cp genome sequences was performed using MAFFT v.7 with the default settings and adjusted manually where necessary with BioEdit v.7.2.5. After alignment, the coding sequences (CDS) were extracted using Geneious v.2019.1.3 [39]. GC content and codon information were calculated with MEGA v.10.0.4.

### Genetic analyses

Two datasets [the whole cp genome and CDS] were used to construct the phylogenetic topology of 14 *Salvia* species with maximum parsimony (MP) and maximum likelihood (ML) methods, respectively. *Mentha longifolia* (NC032054) and *Perilla frutescens* (NC030756) were used as outgroups. The evolutionary divergences of the 14 species were evaluated using nucleotide differences and p-distance by MEGA. Nucleotide diversity and average number of nucleotide differences were calculated by DnaSP v.6.12.03.

### Phylogeographic analyses

To obtain the occurrence records of 14 species, we downloaded natural collection data from the database of Global Biodiversity Information Facility (GBIF, https://www.gbif.org/) and referred to published articles [37, 40, 41]. According to the distribution ranges on the Royal Botanic Gardens (Kew science, http://www.kew.org/science) and literature by Walker et al. [37], we removed duplicate, fuzzy and neighbouring records and further proofread the latitude and longitude with Google Earth [42]. The representative localities of 14 species were marked on the World map to show their geographical distribution.

### Climate analyses

#### Principal component analysis

Ten records per species were chosen randomly using R (v.3.6.3) as target localities to evaluate the influence of spatial climate on TCM substitution. Nineteen WorldClim (v.2.0) bioclimatic layers (see definition in Additional file 1: Table S1) were downloaded from the WorldClim website (https://www.worldclim.org/). Nineteen environmental variables of each locality were extracted with ArcMap v. 10.4 as their ecological dataset (10 localities per species × 14 species × 19 environmental variables). Principal component analysis (PCA) was run with R (v.3.6.3) using the bioclimatic dataset of target localities to examine the relationship between genetic lineages and climatic pattern and discover the ecological similarity of SMSs.

To further understand the ecological processes driving the divergence of pharmacodynamic activities in spite of closely-related species, we performed another PCA using the climate data of (i) *S. miltiorrhiza* and five SMS species (*S. yunnanensis*, *S. prattii, S. bulleyana, S. digitaloide* and *S. przewalskii*); (ii) two nSMS species [*S. plebeia* and *S. roborowskii* (SP&SR)] in Clade3; as well as (iii) *S. deserta*, which was distantly related to *S. miltiorrhiza*, but acted as a folk alternative medicine of Danshen in the west of Xinjiang province.

#### Correlation analyses

To investigate the ecological similarity among SMSs species, we firstly selected two records per species randomly and obtained their climate data (2 localities per species × 14 species × 19 environmental variables, Additional file 1: Table S1 for elaboration) with ArcMap software. Then, Pearson correlation analysis was carried out in R (v.3.6.3) using climate data of each species. A correlation heatmap was created with ggplot2 (v.3.3.3) by using correlation coefficient matrix.

#### Major climate factor analyses

To understand the ecological similarity of the cross-lineages of *Salvia* species, we run MaxEnt (v.3.4.1) [43, 44] three times based on filtered occurrence records. For each time, records in each phylogenetic clade were used to calculate the contribution of each variable to the ecological niche model. Five environmental variables with higher contributions were identified as major climate factors that possessed essential influence on the survival of *Salvia*. The ‘Random test percentage’ was set 25, and ‘Do jackknife to measure variable importance’ was chosen. The remaining parameters were set by default.

### Mantel test

To test the correlation between genetic structure in *Salvia* and the spatial patterning of climate variation within the species range, we performed a partial Mantel test [45] in R by using a Euclidian distance matrix of the climate variables extracted for each locality associated with a genetic sample, compared with the cp genome genetic distance matrix (pairwise uncorrected p) by controlling for geographical distance. We also conducted Mantel test with ade4 (v.1.7-15) and vegan (v.2.5-6) packages in R to compare the Euclidian geographical distance matrix constructed from latitude and longitude for each locality with genetic distance matrix by using 999 permutations.

## Results

### General features of *Salvia* cp genomes

**Fig. 1:**
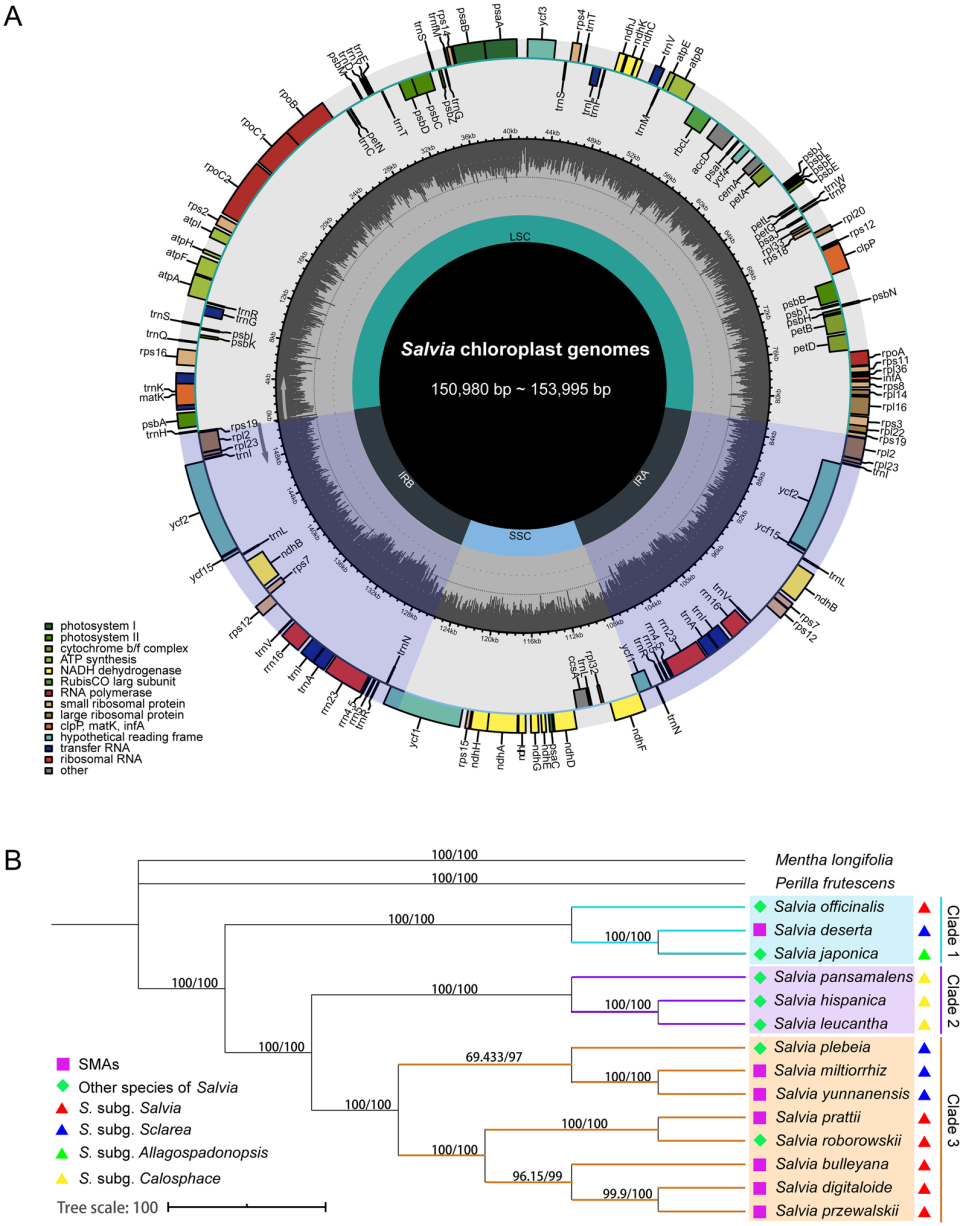
**A** Chloroplast genome schematic map of *Salvia* in this study. The centre of the figure provides length range of the cp genomes. In the first inner circle, the proportion of the shaded parts represents the GC content of each part. The gene names are labelled on the outermost layer. The transcription directions for the inner and outer genes are listed clockwise and anticlockwise, respectively. **B** Phylogenetic relationships of 14 *Salvia* species inferred from MP and ML methods by using complete cp genomes. Numbers above clades are support values with MP bootstrap values on the left and ML bootstrap values on the right. Rose red squares and green diamonds represent *S. miltiorrhiza* and its substitutes as well as other non-substitute species of *Salvia*, respectively

Four cp genomes of *Salvia* species were sequenced, and 22,783,492 to 34,368,080 paired-end raw reads were generated using Illumina Sequencing System. The four novel cp genome sequences have been preserved in GenBank (Table 2). The cp genomes were all circular double-stranded DNA and displayed a quadripartite structure (Additional file 2: Fig. S1, Fig. 1A). The length of the 14 *Salvia* cp genomes ranged from 150,980 to 153,995 bp (Additional file 3: Table S2). For these species, 114 unique coding genes, consisting of 80 protein-coding genes, 30 tRNA genes and four rRNA genes, except for *S. leucantha*, were identically annotated in the same order (Additional file 3: Table S2). The CDS length ranged from 77,064 to 79,455 bp (Additional file 4: Table S3). The number of codons of *S. leucantha* was the least (25,688), while the number of *S. japonica* was the most (26,485).

### Genetic analyses

Phylogenetic analyses were conducted using two methods with the complete cp genomes dataset. The results identified the same lineages within *Salvia*. The trees based on the CDS dataset showed little discrepancy (Additional files 5 and 6: Figs. S2 and S3). Here, we only presented MP topology based on entire cp genomes, and support values were obtained from MP and ML analyses recorded at the corresponding clades (Fig. 1B). Fourteen species were identically divided into three clades (named Clade 1–3). Among the three clades, SMSs, except for *S. deserta*, belonged to Clade 3, indicating that *S. miltiorrhiza* and its substitutes were related species. *S. deserta*, one Danshen-substitutable species, belonged to Clade 1 distantly related to *S. miltiorrhiza*. Clade 3 also consisted of two genetically closely related but pharmacologically divergent species, namely *S. plebeia* and *S. roborowskii*, in comparison with *S. miltiorrhiza*. The three main lineages within *Salvia* were quite distinct (Table 3). Clade 1 had the largest average number of nucleotide differences (1514) within one lineage. The minimum number of average nucleotide differences cross lineages (2596) was found between Clade 2 and 3. The genetic variation within each lineage was significantly smaller than the overall divergent between any two lineages (Table 3).

**Table 3:** Genetic diversity measured for phylogenetic lineages

Clade	n	Number of nucleotides excluding alignment gaps	Pi (π)	k
Clade 1	3	149,660	0.01022	1514.333
Clade 2	3	150,771	0.00221	327.333
Clade 3	8	149,506	0.00340	500.893
Between Clade 1 and Clade 2	6	148,185	0.01723	3641.667
Between Clade 1 and Clade 3	11	147,188	0.01172	3177.875
Between Clade 2 and Clade 3	11	148,091	0.00951	2596.000

Pi (π) is the nucleotide diversity per site; k is the average number of nucleotide differences

### Phylogeographic analyses

**Fig. 2:**
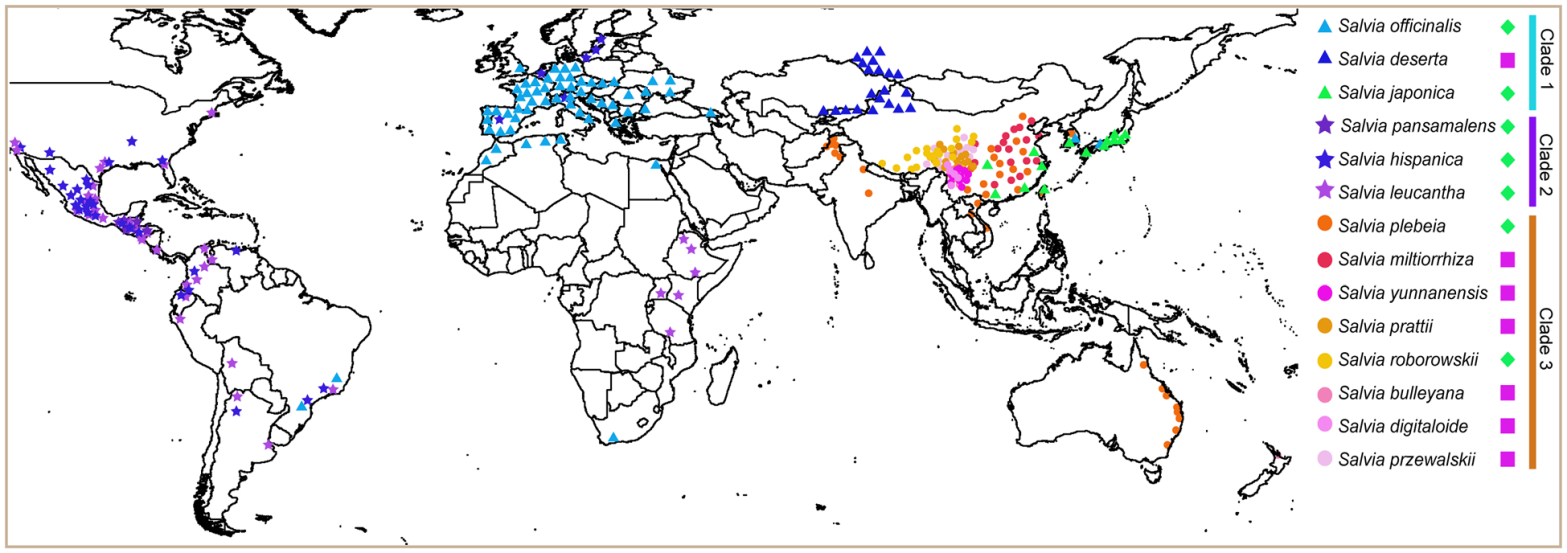
Distribution of 14 *Salvia* species. Rose red squares and green diamonds represent SMSs and nSMSs species studied in this article, respectively. Triangle, circle and pentagram represent such representative occurrence localities of each species. Clade 1–3 are the three lineages of *Salvia* as inferred from phylogenetic analyses

After filtering, 21,160 collection records were preserved. We tagged the representative points of each species on the World map and found that 14 *Salvia* species underwent obvious species radiation in three centres of the world including central and south America, central Asia/Mediterranean and eastern Asia (Fig. 2). The phylogenetic break was almost congruent with that of the geographical distribution of the 14 *Salvia* species. Species in Clade 1, except for *S. japonica*, were mainly distributed in central Asia/Mediterranean region, Clade 2 was mostly distributed in Central and South America, and Clade 3 was chiefly found in eastern Asia, showing obvious phylogeographic structure within their habitat. Among the suitable growing-places of *Salvia*, *S. miltiorrhiza* and its alternatives predominantly settled on eastern and central Asia (Fig. 2), showing a close geographical distance among SMSs. However, two species (SP&SR) were mainly distributed in eastern Asia and closely related to *S. miltiorrhiza*, but their biological activities were distinct from SMSs.

### Climatic analyses

#### PCA analysis

**Fig. 3:**
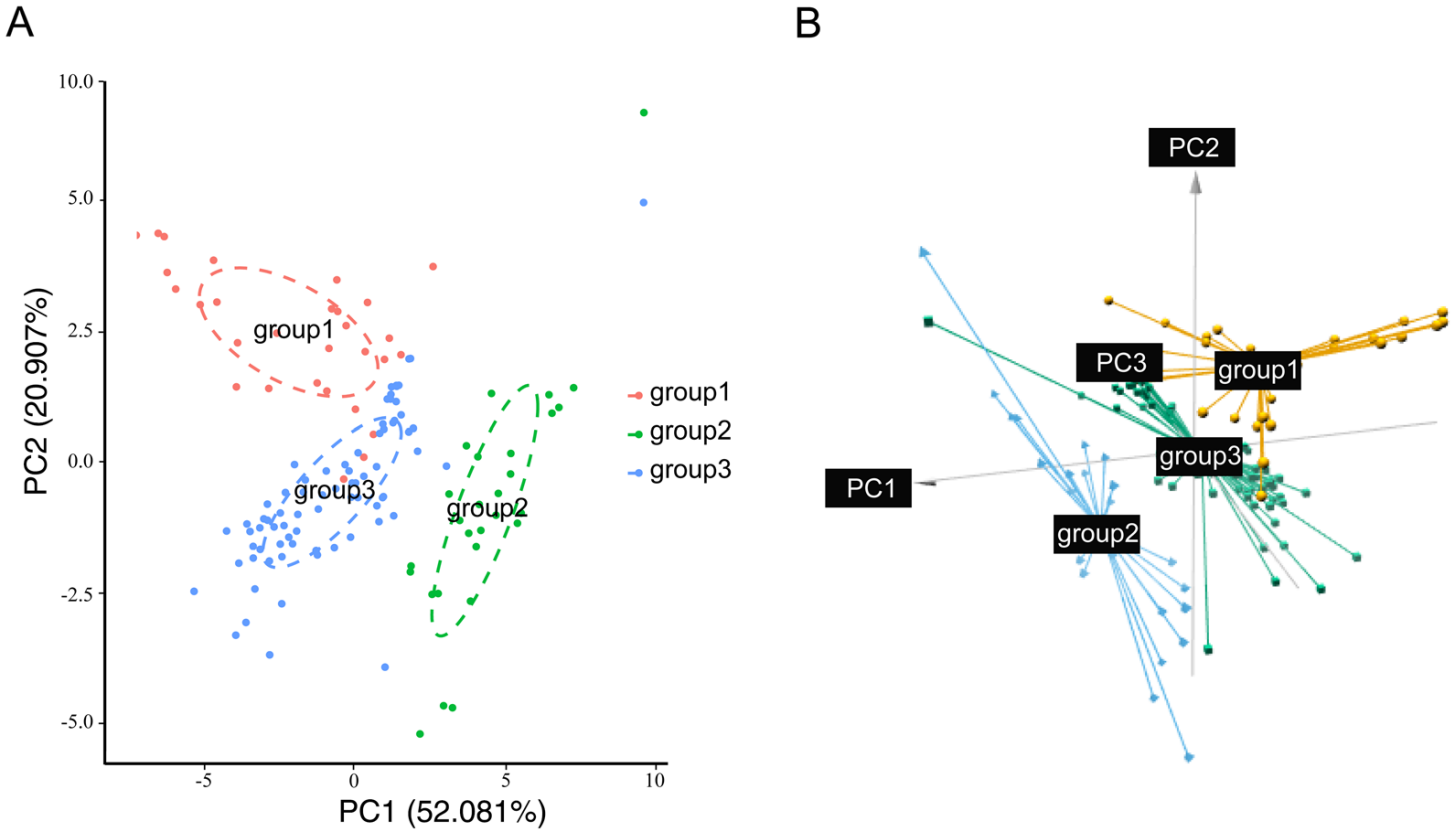
Results of PCA using 2D (**A**) and 3D (**B**) representation. Each point represents the climatic data of each occurrence locality. Different groups are shown with specific colours

According to PC1 (52.081%) and PC2 (20.907%; Fig. 3), PCA plot was obtained. 140 bioclimatic points of 14 species were divided into three well-defined groups (named group 1–3, Fig. 3). Points from group 1 (red circles) were derived from species in Clade 1, group 2 (green circles) corresponded to Clade 2 and group 3 (blue circles) from Clade 3. The climate data grouping of the 14 species are consistent with their genetic lineage structure. *S. miltiorrhiza* and its substitutes, except for *S. deserta*, belonged to group 3, indicating that SMSs were species with a similar growth environment.

**Fig. 4:**
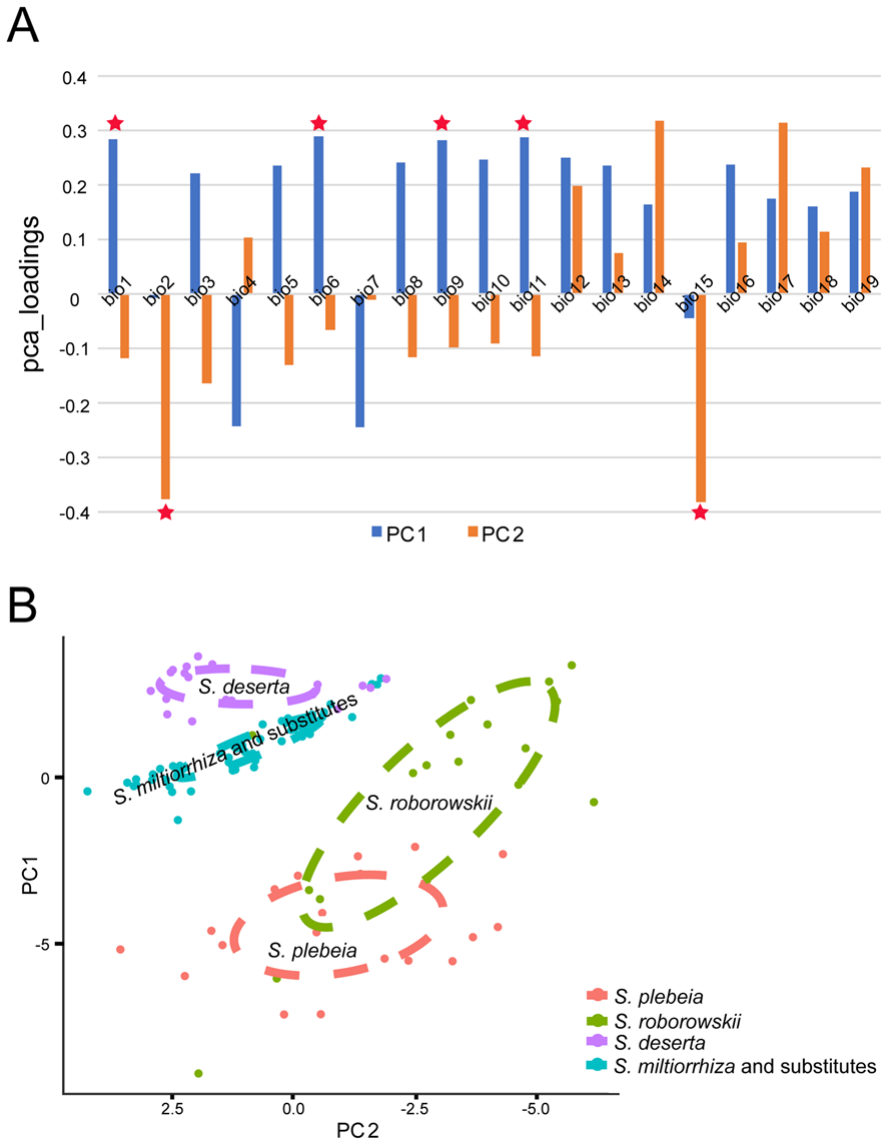
**A** Loading result of the first two principal components. Red star represents climate factors with high loading values in first PCA. **B** Plot of second PCA using climate data of *S. plebeia*, *S. roborowskii*, *S. deserta*, *S. miltiorrhiza* and SMSs

The separation of group 2 from the two other groups was arranged according to PC1, which represented significant differences in bio1, bio6, bio9 and bio11 (Fig. 4A). This finding indicates that environmental differences between group 2 and other groups were mainly linked to temperature, especially winter temperature in the northern hemisphere. PC2 separated group 1 from the two other groups (Fig. 3). PC2 could be primarily described as the environmental variables of bio2 and bio15 (Fig. 4A), indicating that differential climate factors between group 1 and other *Salvia* species mostly lied in the changing ranges of temperature and precipitation.

After combining the phylogenetic relationships and alternative characteristics of *Salvia* species, we found three exceptions including related but non-substitutable *S. plebeia* and *S. roborowskii* as well as relatively distant but substitutable *S. deserta* (Fig. 1B). To explore whether the substitution of the three species was affected by climate, we performed another PCA and compared their ecological similarity to *S. miltiorrhiza* and substitutes. The plot of the first two components explaining the highest percentage of variance (cumulative proportion 64.43%) showed the separation of *S. plebeia*, *S. roborowskii* and *S. deserta* from *S. miltiorrhiza* and SMSs (Fig. 4B). Compared with *S. plebeia* and *S. roborowskii*, the cluster of *S. deserta* was closer to that of *S. miltiorrhiza* and SMSs with few overlapping points.

#### Correlation analyses

**Fig. 5:**
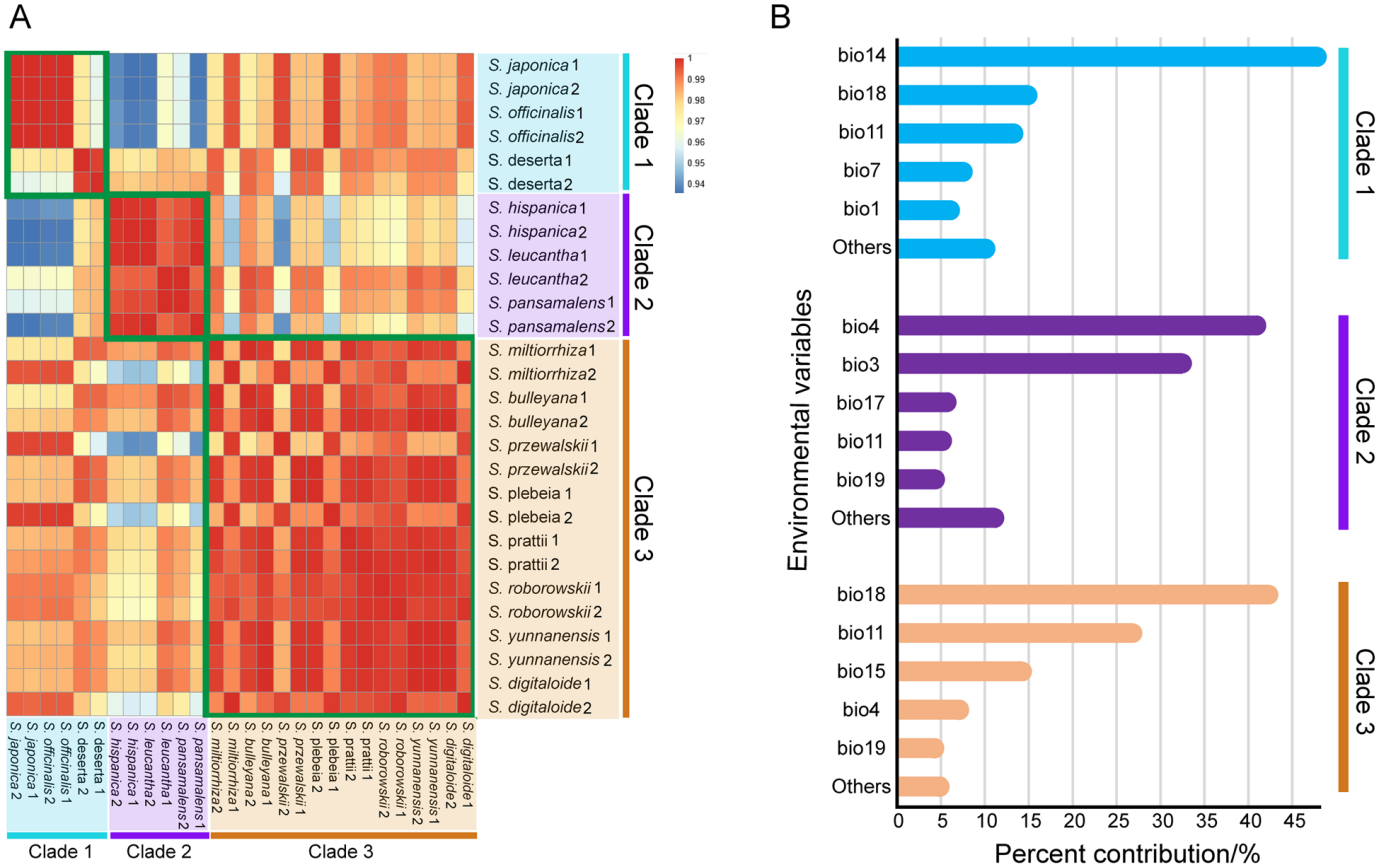
Results of checking ecological similarity of *Salvia* species. **A** Correlation heatmap of growth environments of 14 *Salvia* species. Clade 1–3 represent three lineages inferred from phylogenetic analyses. **B** Jackknife test for variable importance in the suitability distribution of *Salvia*. Values shown were averages of 10 replicate runs

The environmental variables of the 14 *Salvia* species were greatly correlated with Pearson correlation coefficient ranging from 0.943 to 0.997 (Fig. 5A). The habitat similarity within each clade was apparently higher than that between two clades, especially for Clade 2 and 3. The ecological similarity of species within Clade 3 which was mainly composed of SMSs according to phylogenetic analyses was significantly higher than that between Clade 3 and the two other clades.

#### Major climate factor analyses

In the maxent model, the mean Area Under Curve values of 10 replicates of the training and test data were 0.951 and 0.994 for Clade 1, 0.976 and 0.992 for Clade 2 and 0.972 and 0.987 for Clade 3, respectively. The accuracy of the model was ‘excellent’ [46]. Figure 5B shows the importance of environmental variables to the survival of each clade according to jackknife test. The major climate factors of each clade were quite distinct (Fig. 5B). For Clade 1, the major climate factors contained bio14 [precipitation of driest month; percent contribution (pc) the highest 47.9%], bio18, bio11, bio7 and bio1. The major climate factors of Clade 2 were bio4 (temperature seasonality; pc the highest 41%), bio3, bio17, bio11 and bio19. The major climate factors of Clade 3 were bio18 (precipitation of warmest quarter of the year; pc the highest 42.4%), bio11, bio15, bio4 and bio19. Among three clades, the major climate factors of Clade 1 and 3 were relatively similar and both linked to precipitation.

### Mantel test

Partial Mantel test was conducted to examine the relationship of environmental distance and genetic distance while controlling for geographical distance, and the result was significant (r = 0.4659; *P* = 0.002). The Mantel test showed that the genetic structure of *Salvia* was also driven by geographical isolation (r = 0.4903, *P* = 0.012 with ‘ade4’ package; r = 0.4286, *P* = 0.003 with ‘vegan’ package).

## Discussion

In contrast to adulterations, substitution can be legitimate in Foster’s practical definition [4]. Substitution involves offering substances in place of other more expensive ingredients or substituting substances for others that might not be readily available or available only at higher price [47]. Accordingly, in the case of some traditional medicines originated from endangered species or with huge consumption in the market, substitution could be performed by local folks even though Chinese Pharmacopoeia has legally provided sources of each medicinal material. For instance, Astragali Radix (Huangqi), the dried root of *Astragalus membranaceus* (Fisch.) Bge. or *A. membranaceus* var. *Mongholicus* (Bge.) [48], is frequently substituted by Hedysari Radix (Hongqi), the root of *Hedysarum polybotrys*, in Gansu province of China [49]. With regard to *Salvia*, more than 20 congeneric species can be used as substitutes for *S. miltiorrhiza* by local folk [11]. For instance, *S. przewalskii*, *S. yunnanensis* and *S. trijuga* are three species grown in the southwest of China, and their roots are commonly used as Danshen in practice [50]. Meanwhile, many other *Salvia* species exist, and they have application or efficacy distinct from *S. miltiorrhiza*. For example, the whole plant of *S. plebeia* principally has anti-inflammatory, antioxidative, antibacterial and antiviral activities, excluding anti-thrombotic effects [13]. In this study, we took the genus *Salvia* for example, and candidate species were selected predominantly based on the summary by Xiao et al. [12] to explore vital influences determining which species could be used as *S. miltiorrhiza* substitutes.

To date, there have been some scientific reports associated with TCM substitutions mainly focusing on comparison of chemical compounds/metabolism and pharmacological effects between licensed originals and substitutes. Zhang et al. [51] compared the metabolite compositions of *Ophiocordyceps sinensis* and its substitute cultured fermentation mycelia. They found that natural *O. sinensis* and its substitute showed significant differences in their metabolic profiles. BUT et al. [52] provided strengthened evidence that water buffalo horn could be used as an alternative for rhinoceros horn for its purging heat activities. For *Salvia*, Xiao et al. [12] made a comprehensive summary regarding the characteristics of chemical compounds between SMSs and nSMSs, and reported that lipophilic diterpene, one active ingredient of Danshen, was relatively higher in SMSs species. However, minor or even only traces were found in nSMSs. Several other investigations [53–56] reported similar observations in *Salvia*. These studies did not clearly specify substitute delimitation owing to ambiguous fundamental influential factors to determine substitutes in medicinal practice. Theories involving chemical composition and pharmacodynamics could not guide the discovery of alternative resources.

Pharmaphylogeny is a new frontier subject that interrogates the phylogenetic relationship of medicinal plants as well as the intrinsic correlation of molecular phylogeny, chemical constituents and therapeutic efficacy [57, 58]. The theory of pharmaphylogeny suggests that species with genetic links should be preferentially chosen as candidate substitutes and emphasizes the important role of genetic relationships in the development of surrogate resources [50]. This theory has guided many successful cases of new medicinal resource exploitation. For example, *Picroihiza scrophulariiflora* (Xizang Huanglian) was discovered to be a substitute for *P*. *kurrooa* Royle ex Benth. (Huhuanglian) originated from India of Rhizoma Picrorhizae in the Chinese Pharmacopoeia 1995 edition [59]. However, in resource exploitation, some plant medicines whose related species were found in China had no pharmacological effects similar to target medicines [30]. Hence, other factors, apart from phylogenetics, could influence alternative species selection.

Basing on the scientific elucidation of daodi medicinal materials [31, 32], we suspected that ecology might be another key factor and hypothesized that phylogenetic relationship and geographical climate work together to determine which *Salvia* species has the potential to be selected as substitutes for *S. miltiorrhiza*. We tested this hypothesis from phylogenetic and ecological perspectives. Firstly, we took advantage of 14 cp genomes to conduct phylogenetic analysis since single or multiple DNA barcodes could not perfectly distinguish intraspecific and interspecific variation of *Salvia *[60, 61]. The 14 *Salvia* species came from all three distribution centres [37], of which three species were predominantly distributed in central Asia/Mediterranean, eight species were distributed in eastern Asia, and three species were distributed in central and south America. These species could cover different natural ecological environment of *Salvia*. Three main lineages were found in this topology of *Salvia*, congruent with that obtained by Walker et al. [62] and Hu et al. [63]. *S. miltiorrhiza* and SMSs were predominantly located in Clade 3 (Fig. 1B), which implied that *S. miltiorrhiza* and alternative species were closely related to each other. This result is consistent with the view of Pharmaphylogeny. Phylogeny is indeed an important influential factor that determines substitution. Secondly, we interestingly found that (i) *S. plebeia* and *S. roborowskii* were genetically related to *S. miltiorrhiza* but had distinct clinical applications (Table 1). (ii) Although *S. deserta* was in Clade1 relatively distantly related to *S. miltiorrhiza* (Fig. 1B), it had medicinal effects similar to those of *S. miltiorrhiza* and could be used as an alternative in Xinjiang region. The existence of three exceptional species suggested that other factors, apart from phylogeny, could influence substitution.

To test the role of ecology in alternative species selection, we carried out a series of analyses including PCA (Figs. 3 and 4B), correlation (Fig. 5A) and major climate factor analyses (Fig. 5B). In PCA, the climate data of 14 species were well separated into three groups (Fig. 3). The bioclimatic points of *S. miltiorrhiza* and SMSs species gathered for a group imply their similar habitat. The growth environment of *S. plebeia* and *S. roborowskii* was well separated from that of *S. miltiorrhiza* and SMSs based on the second PCA (Fig. 4B). This finding indicated that their efficacy differentiation and non-fungible property could be attributed to environmental differences. Few overlap-points were found between *S. deserta* as well as *S. miltiorrhiza* and SMSs group (Fig. 4B), confirming the impact of ecology on determining *S. miltiorrhiza* substitutes. The result of the correlation analyses (Fig. 5A) suggested that the climate of species within each clade was apparently similar to that between two clades. *S. miltiorrhiza* and SMSs species were primarily located within Clade 3, and their growth environment were quite similar. To further confirm the classification of the habitat in *Salvia*, we conducted major climate factor analysis using thorough collection records. The major climate factors of three phylogenetic lineages were distinct (Fig. 5B). However, the major climate factors of Clade 1 and 3 were bio14 and bio18, which were both related to precipitation, in spite of being in different periods. Hence, the growth environment of species in Clades 1 and 3 was somewhat similar and could explain why *S. deserta* in Clade 1 could be a substitute for *S. miltiorrhiza* to some extent. Thus far, all the three analyses proved the role of ecology in determining substitution for *S. miltiorrhiza*. Besides, inferred from Fig. 2, the ecological differences between ‘*S. miltiorrhiza* and SMSs’ and nSMSs species in this study were originated from different geographical distribution. So here, two factors of ecology and geographical distribution of *Salvia* can be integrated in a comprehensive way.

By combining the present results with relevant reports [64, 65], we argued that phylogenetic relationship and environmental stress could be two forces that contribute to the delimitation of substitutable species and the homogeneity and diversity of medicinal effects of species within one genus. Genetics fundamentally affects the type of bioactive components and biosynthetic and pharmacological function. *S. miltiorrhiza* and substitutes are used interchangeably in folk, reflecting the similarity in the type of bioactive ingredients and their chemical profile. This similarity could be attributed to the shared genetic base associated with synthesis of two main active compounds (phenolic acids and lipophilic diterpene components), metabolic pathways and accumulation pattern [66]. For another, environmental signals, as (i) abiotic elicitors that influence plant secondary metabolism [67] and (ii) motive forces that provide directions for genetic variation and evolution [68], could be important regulators that lead to similar efficacy between *S. miltiorrhiza* and substitutes. This study presented the natural biology of medicinal resource substitution. Other medicinal plants can learn from this biology towards developing alternative resources.

In this study, two main influences of both phylogeny and ecology were focused when studying the biology of medicinal resource substitution, of course, we realized that some other factors, such as medication customs, market accounts [69], medical culture, socioeconomic [70] and species distribution, may also have an impact on substitution. European and American distribution centre countries use more chemicals than herbs. In economically developed areas, the choice of medicine was wider, so it would not be limited to the discovery of alternative species [71]. The content of lipophilic diterpene, one active ingredient in Danshen, was minor or even only traces in species mainly located in Europe and America, which could also lead to their non-substitution for *S. miltiorrhiza*. The alternative character of *S. deserta* could be attributed to the shape of similar growth environment, inferred from our study (Figs. 3, 4 and 5), as well as to its available local genetic material in western Chinese region Xinjiang. Therefore, this study only revealed the biology of resource substitution of *S. miltiorrhiza* from the perspective of natural science. Besides, we do note that in this study, there was a limitation of cp genomes and as a consequence, it might not fully show the real effect of phylogeny and ecology on substitution. However, our sampling size is by far the largest and most extensive in medicinal resource substitution research. Additional *Salvia* cp genomes and bioclimatic data will be needed to improve our ability to examine and better define the role of phylogeny and ecology in resource substitution in the following research.

## Conclusions

At present, no in-depth attempts have been conducted to study vital determinants, although many herbal medicines have been in demand for resource substitution. This study is the first to focus on crucial influential factors of determining substitutes in TCM from the perspective of phylogeny and ecology. Phylogenetic relationship and geographical climate were two fundamental elements working together to shape substitutes for *S. miltiorrhiza*. Homogeneity and diversity of medicinal efficacy of related species could be attributed to similarities and differentiations of genetic and ecological type observed in *Salvia*. This study would benefit us by targeting candidate substitutes accurately and further simplifying screening processes. Although total sampling remains small, we believe that these species can reflect the genetic relationship and ecological types of the three natural distribution areas of the genus *Salvia*. Our study could enrich the content of pharmaphylogeny by adding another consideration of ecology, especially climate, promote the rationality of substitution and provide guidance for TCM introduction and new resource development.

## Supplementary Information

**Additional file 1: Table S1.** Definition of nineteen bioclimatic factors.

**Additional file 2: Figure S1. **Chloroplast genome maps of *S. deserta* (A), *S. digitaloides* (B), *S. leucantha* (C), and *S. pansamalensis* (D). The genes inside and outside of the circle were transcribed in the clockwise and counterclockwise directions, respectively.

**Additional file 3: Table S2.** Summary statistics of the 14 *Salvia* chloroplast genomes.

**Additional file 4: Table S3. **Features of CDS sequences in fourteen *Salvia* species.

**Additional file 5: Figure S2. **Phylogenetic relationships of the 14 *Salvia* species inferred from maximum parsimony (MP) analysis of CDS regions. Numbers above clades are MP bootstrap values.

**Additional file 6: Figure S3. **Phylogenetic relationships of the 14 *Salvia* species inferred from maximum likelihood (ML) analysis of CDS regions. Numbers above clades are ML bootstrap values.

## Data Availability

The chloroplast genome sequences assembled in this study are openly available in NCBI (https://www.ncbi.nlm.nih.gov) GenBank with the Accession numbers of MT156378, MT156367, MT156368 and MT156376. Raw sequencing data is accessible at NCBI SRA database with Accession numbers of SRR14027992, SRR14028488, SRR14038941, and SRR14039176.

## Abbreviations

SMSs: * Salvia miltiorrhiza* substitutes
nSMSs: Non-substitutes for *Salvia miltiorrhiza*
ML: Maximum likelihood
MP: Maximum parsimony
CDS: Coding sequences
TCM: Traditional Chinese medicine
AR: Astragali Radix
cp: Chloroplast
PCA: Principal component analysis
SP&SR: *S. plebeia* and *S. roborowskii*

## References

[1] Tankeu S, Vermaak I, Chen W, Sandasi M, Viljoen A (2016). Differentiation between two “fang ji” herbal medicines, *Stephania tetrandra* and the nephrotoxic *Aristolochia fangchi*, using hyperspectral imaging. Phytochemistry.

[2] Huang H (2011). Plant diversity and conservation in China: planning a strategic bioresource for a sustainable future. Bot J Linn Soc.

[3] Cordell GA (2015). Ecopharmacognosy and the responsibilities of natural product research to sustainability. Phytochem Lett.

[4] Cheung H, Wang Y, Biggs D (2018). China’s reopened rhino horn trade. Science.

[5] Liu R, Wang M, Duan JA, Guo JM, Tang YP (2010). Purification and identification of three novel antioxidant peptides from Cornu Bubali (water buffalo horn). Peptides.

[6] Chen W, Chen G (2017). Danshen (Salvia miltiorrhiza Bunge): a prospective healing sage for cardiovascular diseases. Curr Pharm Des.

[7] Commission CP (2015). Pharmacopoeia of the People’s Republic of China.

[8] Tang J, Xue Z, Daroch M, Ma J (2014). Impact of continuous *Salvia miltiorrhiza* cropping on rhizosphere actinomycetes and fungi communities. Ann Microbiol.

[9] He C-e, Wei J, Jin Y, Chen S (2010). Bioactive components of the roots of Salvia miltiorrhizae: changes related to harvest time and germplasm line. Ind Crop Prod.

[10] Kum KY, Kirchhof R, Luick R, Heinrich M (2021). Danshen (*Salvia miltiorrhiza*) on the global market: what are the implications for products’ quality?. Front Pharmacol.

[11] Li MH, Chen JM, Peng Y, Wu Q, Xiao PG (2008). Investigation of Danshen and related medicinal plants in China. J Ethnopharmacol.

[12] Xiao XH, Fang QM, Xia WJ, Yin GP, Su ZW, Qiao CZ (1997). Numerical taxonomy of medicinal *Salvia* L. and the genuineness of Danshen. J Plant Res Environ.

[13] Liang YY, Wan XH, Niu FJ, Xie SM, Guo H, Yang YY, Guo LY, Zhou CZ (2020). *Salvia plebeia* R. Br.: an overview about its traditional uses, chemical constituents, pharmacology and modern applications. Biomed Pharmacother.

[14] Tabanca N, Demirci B, Turner JL, Pounders C, Demirci F, Baser KH, Wedge DE (2010). Microdistillation and analysis of volatiles from eight ornamental Salvia taxa. Nat Prod Commun.

[15] Shimizu T, Inoue T, Mizuno M (1994). [Historical and herbalogical studies on coloring crude drug (Part 3) “Shu wei cao”]. Yakushigaku Zasshi.

[16] Mocan A, Babota M, Pop A, Fizesan I, Diuzheva A, Locatelli M, Carradori S, Campestre C, Menghini L, Sisea CR, Sokovic M, Zengin G, Paltinean R, Badarau S, Vodnar DC, Crisan G (2020). Chemical constituents and biologic activities of Sage species: a comparison between *Salvia officinalis* L., *S. glutinosa* L. and *S. transsylvanica* (Schur ex Griseb. &amp; Schenk) Schur. Antioxidants (Basel).

[17] Kasimu R, Wang X, Wang X, Hu J, Wang X, Mu Y (2018). Antithrombotic effects and related mechanisms of Salvia deserta Schang root EtOAc extracts. Sci Rep.

[18] Laparra Llopis JM, Brown D, Saiz B (2020). Chenopodium quinoa and Salvia Hispanica provide immunonutritional agonists to ameliorate hepatocarcinoma severity under a high-fat diet. Nutrients.

[19] Li LW, Qi YY, Liu SX, Wu XD, Zhao QS (2018). Neo-clerodane and abietane diterpenoids with neurotrophic activities from the aerial parts of Salvia leucantha Cav. Fitoterapia.

[20] Bisio A, Damonte G, Fraternale D, Giacomelli E, Salis A, Romussi G, Cafaggi S, Ricci D, De Tommasi N (2011). Phytotoxic clerodane diterpenes from Salvia miniata Fernald (Lamiaceae). Phytochemistry.

[21] Cui N, Liao BS, Liang CL, Li SF, Zhang H, Xu J, Li XW, Chen SL (2020). Complete chloroplast genome of *Salvia plebeia*: organization, specific barcode and phylogenetic analysis. Chin J Nat Med.

[22] Li C, Liu Y, Gao Y, Zhang C (2005). [Studies on chemical constituents from Salvia roborowskii Maxim]. Zhong Yao Cai.

[23] Lin YS, Peng WH, Shih MF, Cherng JY (2020). Anxiolytic effect of an extract of Salvia miltiorrhiza Bunge (Danshen) in mice. J Ethnopharmacol.

[24] Huang C, Chen KL (2007). [Contrast studies on content of hydrophobic components between Salvia yunnanensis roots and Salvia miltiorrhiza roots]. Zhong Yao Cai.

[25] Xia F, Li WY, Yang XW, Yang J, Li X, Nian Y, Xu G (2019). Salpratlactones A and B: a pair of cis-trans tautomeric abietanes as Cav3.1 T-type calcium channel agonists from *Salvia prattii*. Org Lett.

[26] Grzegorczyk-Karolak I, Krzeminska M, Kiss AK, Olszewska MA, Owczarek A (2020). Phytochemical profile and antioxidant activity of aerial and underground parts of *Salvia bulleyana* Diels. plants. Metabolites.

[27] Xu G, Yang J, Wang YY, Peng LY, Yang XW, Pan ZH, Liu ED, Li Y, Zhao QS (2010). Diterpenoid constituents of the roots of Salvia digitaloides. J Agric Food Chem.

[28] Wang Y, Duo D, Yan Y, He R, Wang S, Wang A, Wu X (2020). Bioactive constituents of Salvia przewalskii and the molecular mechanism of its antihypoxia effects determined using quantitative proteomics. Pharm Biol.

[29] Hao DC, Xiao PG, Liu LW, Peng Y, He CN (2015). [Essentials of pharmacophylogeny: knowledge pedigree, epistemology and paradigm shift]. Zhongguo Zhong Yao Za Zhi.

[30] Tang GX, Dan Y (2016). The establishment of pharmaphylogeny. Sci Cult Rev.

[31] Zhao Z, Guo P, Brand E (2012). The formation of daodi medicinal materials. J Ethnopharmacol.

[32] Liu X, Zhang Y, Wu M, Ma Z, Huang Z, Tian F, Dong S, Luo S, Zhou Y, Zhang J, Li N, He X, Cao H (2020). The scientific elucidation of daodi medicinal materials. Chin Med.

[33] Liang CL, Wang L, Lei J, Duan BZ, Ma WS, Xiao SM, Qi H, Wang Z, Liu Y, Shen X, Guo S, Hu H, Xu J, Chen S (2019). A comparative analysis of the chloroplast genomes of four salvia medicinal plants. Engineering.

[34] Qian J, Song J, Gao H, Zhu Y, Xu J, Pang X, Yao H, Sun C, Li X, Li C, Liu J, Xu H, Chen S (2013). The complete chloroplast genome sequence of the medicinal plant *Salvia miltiorrhiza*. PLoS ONE.

[35] Wu J, Li X, Huang L, Meng X, Hu H, Luo L, Chen S (2019). A new GIS model for ecologically suitable distributions of medicinal plants. Chin Med.

[36] Shen L, Li XW, Meng XX, Wu J, Tang H, Huang LF, Xiao SM, Xu J, Chen SL (2019). Prediction of the globally ecological suitability of *Panax quinquefolius* by the geographic information system for global medicinal plants (GMPGIS). Chin J Nat Med.

[37] Walker JB, Sytsma KJ, Treutlein J, Wink M (2004). Salvia (Lamiaceae) is not monophyletic: implications for the systematics, radiation, and ecological specializations of Salvia and tribe Mentheae. Am J Bot.

[38] Lohse M, Drechsel O, Kahlau S, Bock R (2013). OrganellarGenomeDRAW—a suite of tools for generating physical maps of plastid and mitochondrial genomes and visualizing expression data sets. Nucleic Acids Res.

[39] Kearse M, Moir R, Wilson A, Stones-Havas S, Cheung M, Sturrock S, Buxton S, Cooper A, Markowitz S, Duran C, Thierer T, Ashton B, Meintjes P, Drummond A (2012). Geneious basic: an integrated and extendable desktop software platform for the organization and analysis of sequence data. Bioinformatics.

[40] Zhong GX, Li P, Zeng LJ, Guan J, Li DQ, Li SP (2009). Chemical characteristics of *Salvia miltiorrhiza* (Danshen) collected from different locations in China. J Agric Food Chem.

[41] Wang YL, Hao JD, Li MH (2016). [Herbal textural and original plants research on medicines from Salvia in China]. Zhongguo Zhong Yao Za Zhi.

[42] Lal P, Prakash A, Kumar A (2020). Google Earth Engine for concurrent flood monitoring in the lower basin of Indo-Gangetic-Brahmaputra plains. Nat Hazards (Dordr).

[43] Melo-Merino SM, Reyes-Bonilla H, Lira-Noriega A (2020). Ecological niche models and species distribution models in marine environments: a literature review and spatial analysis of evidence. Ecol Model.

[44] Wang R, Yang H, Luo W, Wang M, Lu X, Huang T, Zhao J, Li Q (2019). Predicting the potential distribution of the Asian citrus psyllid, Diaphorina citri (Kuwayama), in China using the MaxEnt model. PeerJ.

[45] Gu B, Wang Y, Xu J, Jiao N, Xu D (2021). Water mass shapes the distribution patterns of planktonic ciliates (Alveolata, Ciliophora) in the subtropical Pearl River Estuary. Mar Pollut Bull.

[46] Jiang H, Liu T, Li L, Zhao Y, Pei L, Zhao J (2016). Predicting the potential distribution of *Polygala tenuifolia* Willd. under climate change in China. PLoS ONE.

[47] Foster S (2011). A brief history of adulteration of herbs, spices, and botanical drugs. HerbalGram.

[48] Gao Z, Liu Y, Wang X, Song J, Chen S, Ragupathy S, Han J, Newmaster SG (2017). Derivative technology of DNA barcoding (Nucleotide Signature and SNP Double Peak Methods) detects adulterants and substitution in Chinese patent medicines. Sci Rep.

[49] Liu J, Hu X, Yang Q, Yu Z, Zhao Z, Yi T, Chen H (2010). Comparison of the immunoregulatory function of different constituents in radix astragali and radix hedysari. J Biomed Biotechnol.

[50] Hao DC, Gu XJ, Xiao PG. Phytochemical and biological research of Salvia medicinal resources. In: Medicinal plants. 2015. p. 587–639.

[51] Zhang J, Zhong X, Li S, Zhang G, Liu X (2015). Metabolic characterization of natural and cultured *Ophicordyceps sinensis* from different origins by 1H NMR spectroscopy. J Pharm Biomed Anal.

[52] But PP, Lung LC, Tam YK (1990). Ethnopharmacology of rhinoceros horn. I: antipyretic effects of rhinoceros horn and other animal horns. J Ethnopharmacol.

[53] Wang T, Zhang H, Wang L, Jiang Y, Zhang L, Zhou Y, Yang R, Ding C, Wang X (2014). A simple and reliable method for distinguishing danshen in salvia: simultaneous quantification of six active compositions by HPLC. J Chromatogr Sci.

[54] Dang J, Cui Y, Pei J, Yue H, Liu Z, Wang W, Jiao L, Mei L, Wang Q, Tao Y, Shao Y (2018). Efficient separation of four antibacterial diterpenes from the roots of salvia prattii using non-aqueous hydrophilic solid-phase extraction followed by preparative high-performance liquid chromatography. Molecules.

[55] Poulios E, Giaginis C, Vasios GK (2019). Current advances on the extraction and identification of bioactive components of Sage (*Salvia* spp.). Curr Pharm Biotechnol.

[56] Ghorbani A, Esmaeilizadeh M (2017). Pharmacological properties of *Salvia officinalis* and its components. J Tradit Complement Med.

[57] Hao DC, Xiao PG, Liu M, Peng Y, He CN (2014). [Pharmaphylogeny vs. pharmacophylogenomics: molecular phylogeny, evolution and drug discovery]. Yao Xue Xue Bao.

[58] Gong X, Yang M, He CN, Bi YQ, Zhang CH, Li MH, Xiao PG. Plant pharmacophylogeny: review and future directions. Chin J Integr Med. 2020. 10.1007/s11655-020-3270-9 33170942

[59] Rokaya MB, Parajuli B, Bhatta KP, Timsina B (2020). *Neopicrorhiza scrophulariiflora* (Pennell) Hong: a comprehensive review of its traditional uses, phytochemistry, pharmacology and safety. J Ethnopharmacol.

[60] Drew BT, Sytsma KJ (2011). Testing the monophyly and placement of Lepechinia in the Tribe Mentheae (Lamiaceae). Syst Bot.

[61] Drew BT, Sytsma KJ (2012). Phylogenetics, biogeography, and staminal evolution in the tribe Mentheae (Lamiaceae). Am J Bot.

[62] Walker JB, Sytsma KJ (2007). Staminal evolution in the genus *Salvia* (Lamiaceae): molecular phylogenetic evidence for multiple origins of the staminal lever. Ann Bot.

[63] Hu G, Takano A, Drew BT, Liu E, Soltis DE, Soltis PS, Peng H, Xiang C (2018). Phylogeny and staminal evolution of Salvia (Lamiaceae, Nepetoideae) in East Asia. Ann Bot.

[64] Johnson W (2007). Genetic and environmental influences on behavior: capturing all the interplay. Psychol Rev.

[65] Mosing MA, Pedersen NL, Cesarini D, Johannesson M, Magnusson PK, Nakamura J, Madison G, Ullen F (2012). Genetic and environmental influences on the relationship between flow proneness, locus of control and behavioral inhibition. PLoS ONE.

[66] Yang D, Fang Y, Xia P, Zhang X, Liang Z (2018). Diverse responses of tanshinone biosynthesis to biotic and abiotic elicitors in hairy root cultures of *Salvia miltiorrhiza* and *Salvia castanea* Diels f. tomentosa. Gene.

[67] Zhou M, Memelink J (2016). Jasmonate-responsive transcription factors regulating plant secondary metabolism. Biotechnol Adv.

[68] Rogers DS, Ehrlich PR (2008). Natural selection and cultural rates of change. Proc Natl Acad Sci USA.

[69] Xu J, Wei K, Zhang G, Lei L, Yang D, Wang W, Han Q, Xia Y, Bi Y, Yang M, Li M (2018). Ethnopharmacology, phytochemistry, and pharmacology of Chinese *Salvia* species: a review. J Ethnopharmacol.

[70] Ge S, He TT, Hu H (2014). Popularity and customer preferences for over-the-counter Chinese medicines perceived by community pharmacists in Shanghai and Guangzhou: a questionnaire survey study. Chin Med.

[71] Kamath S, Skeels M, Pai A (2009). Significant differences in alkaloid content of *Coptis chinensis* (Huanglian), from its related American species. Chin Med.

