# Supplementary material for: The biology of medicinal resource substitution in Salvia
Source: Chin Med. 2021 Dec 23;16:141. doi: 10.1186/s13020-021-00548-6 (PMC8705193; doi:10.1186/s13020-021-00548-6)
Supplement: Supplementary file 2 — Additional file 2: Figure S1. Chloroplast genome maps of S. deserta (A), S. digitaloides (B), S. leucantha (C), and S. pansamalensis (D). The genes inside and outside of the circle were transcribed in the clockwise and counterclockwise directions, respectively. [file 13020_2021_548_MOESM2_ESM.docx]

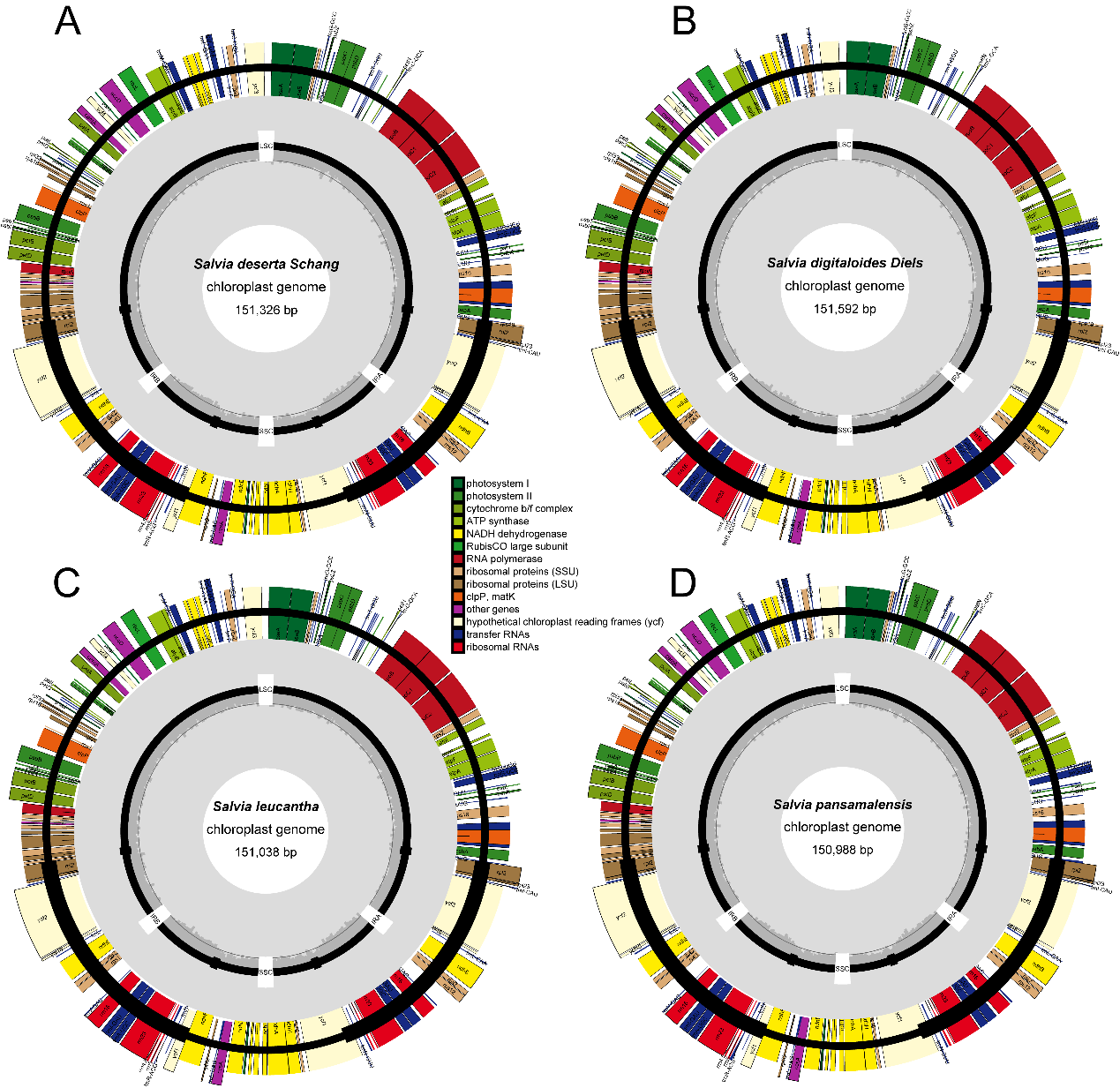


**Figure S1**. Chloroplast genome maps of *S. deserta* (A), *S. digitaloides* (B), *S. leucantha* (C), and *S. pansamalensis* (D). The genes inside and outside of the circle were transcribed in the clockwise and counterclockwise directions, respectively.
