# Supplementary material for: The biology of medicinal resource substitution in Salvia
Source: Chin Med. 2021 Dec 23;16:141. doi: 10.1186/s13020-021-00548-6 (PMC8705193; doi:10.1186/s13020-021-00548-6)
Supplement: Supplementary file 3 — Additional file 3: Table S2. Summary statistics of the 14 Salvia chloroplast genomes. [file 13020_2021_548_MOESM3_ESM.docx]

**Table S2**. Summary statistics of the 14 *Salvia* chloroplast genomes

|  | Species | Total cp genome size (bp) | Number of uniq protein-coding genes | Number of uniq tRNAs | Number of uniq rRNAs |
| --- | --- | --- | --- | --- | --- |
| 1 | *S*. *miltiorrhiza* | 151,328 | 80 | 30 | 4 |
| 2 | *S*. *japonica* | 153,995 | 80 | 30 | 4 |
| 3 | *S*. *bulleyana* | 151,547 | 80 | 30 | 4 |
| 4 | *S*. *officinalis* | 151,089 | 80 | 30 | 4 |
| 5 | *S*. *przewalskii* | 151,319 | 80 | 30 | 4 |
| 6 | *S*. *plebeia* | 151,062 | 80 | 30 | 4 |
| 7 | *S. prattii* | 151,690 | 80 | 30 | 4 |
| 8 | *S. roborowskii* | 151,654 | 80 | 30 | 4 |
| 9 | *S. yunnanensis* | 151,396 | 80 | 30 | 4 |
| 10 | *S. hispanica* | 150,980 | 80 | 30 | 4 |
| 11 | *S. deserta* | 151,326 | 80 | 30 | 4 |
| 12 | *S. digitaloides* | 151,592 | 80 | 30 | 4 |
| 13 | *S. leucantha* | 151,038 | 79 | 30 | 4 |
| 14 | *S. pansamalensis* | 150,988 | 80 | 30 | 4 |
