# Supplementary material for: The biology of medicinal resource substitution in Salvia
Source: Chin Med. 2021 Dec 23;16:141. doi: 10.1186/s13020-021-00548-6 (PMC8705193; doi:10.1186/s13020-021-00548-6)
Supplement: Supplementary file 4 — Additional file 4: Table S3. Features of CDS sequences in fourteen Salvia species. [file 13020_2021_548_MOESM4_ESM.docx]

**Table S3**. Features of CDS sequences in fourteen *Salvia* species

|  | Species | Length of CDS sequences | Number of condons | GC content of CDS sequences (%) |
| --- | --- | --- | --- | --- |
| 1 | *S*. *miltiorrhiza* | 79,449 | 26483 | 38.12 |
| 2 | *S*. *japonica* | **79,455** | **26485** | 38.01 |
| 3 | *S*. *bulleyana* | 79,335 | 26445 | 38.10 |
| 4 | *S*. *officinalis* | 79,389 | 26463 | 38.13 |
| 5 | *S*. *przewalskii* | 79,341 | 26447 | 38.09 |
| 6 | *S*. *plebeia* | **79,293** | **26431** | 38.07 |
| 7 | *S. prattii* | 79,341 | 26447 | 38.13 |
| 8 | *S. roborowskii* | 79,359 | 26453 | 38.10 |
| 9 | *S. yunnanensis* | 79,308 | 26436 | 38.11 |
| 10 | *S. hispanica* | 79,095 | 26365 | 37.99 |
| 11 | *S. deserta* | 79,404 | 26468 | 38.05 |
| 12 | *S. digitaloides* | 79,341 | 26447 | 38.11 |
| 13 | *S. leucantha* | 77,064 | 25688 | 38.18 |
| 14 | *S. pansamalensis* | 79,296 | 26432 | 38.06 |
