# Supplementary material for: The biology of medicinal resource substitution in Salvia
Source: Chin Med. 2021 Dec 23;16:141. doi: 10.1186/s13020-021-00548-6 (PMC8705193; doi:10.1186/s13020-021-00548-6)
Supplement: Supplementary file 5 — Additional file 5: Figure S2. Phylogenetic relationships of the 14 Salvia species inferred from maximum parsimony (MP) analysis of CDS regions. Numbers above clades are MP bootstrap values. [file 13020_2021_548_MOESM5_ESM.docx]

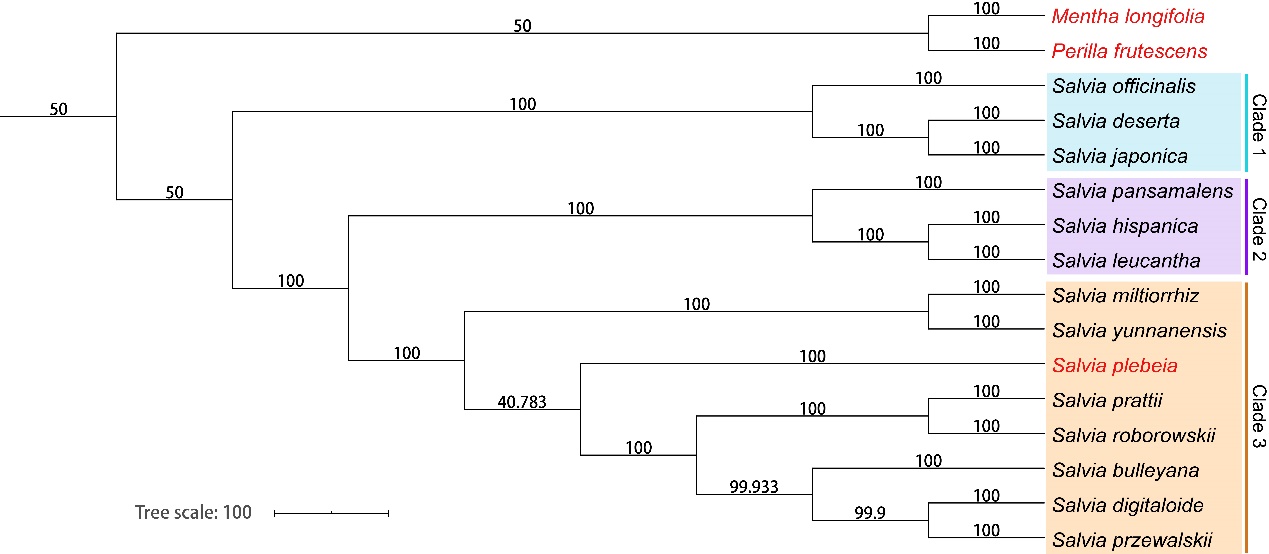


**Figure S2**. Phylogenetic relationships of the 14 *Salvia* species inferred from maximum parsimony (MP) analysis of CDS regions. Numbers above clades are MP bootstrap values.
