# Supplementary material for: The biology of medicinal resource substitution in Salvia
Source: Chin Med. 2021 Dec 23;16:141. doi: 10.1186/s13020-021-00548-6 (PMC8705193; doi:10.1186/s13020-021-00548-6)
Supplement: Supplementary file 6 — Additional file 6: Figure S3. Phylogenetic relationships of the 14 Salvia species inferred from maximum likelihood (ML) analysis of CDS regions. Numbers above clades are ML bootstrap values. [file 13020_2021_548_MOESM6_ESM.docx]

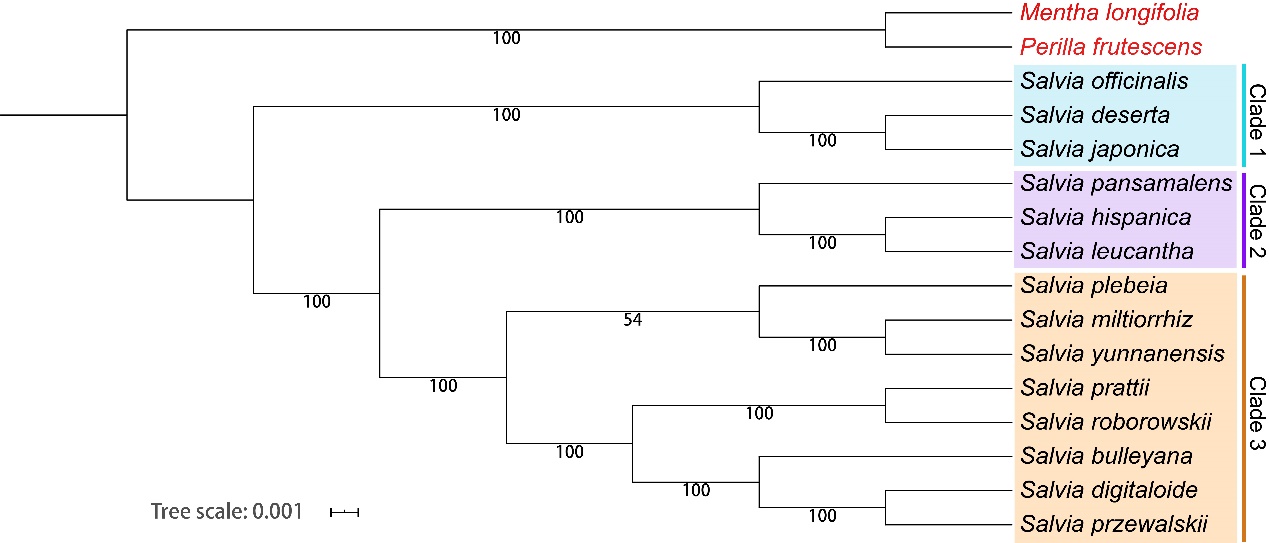


**Figure S3**. Phylogenetic relationships of the 14 *Salvia* species inferred from maximum likelihood (ML) analysis of CDS regions. Numbers above clades are ML bootstrap values.
